# GLFNet: Attention Mechanism-Based Global–Local Feature Fusion Network for Micro-Expression Recognition

**DOI:** 10.3390/e27101023

**Published:** 2025-09-28

**Authors:** Meng Zhang, Long Yao, Wenzhong Yang, Yabo Yin

**Affiliations:** 1 School of Computer Science and Technology, Xinjiang University, Urumqi 830017, China; menka@stu.xju.edu.cn (M.Z.); 107552301321@stu.xju.edu.cn (L.Y.); yinyabo@xju.edu.cn (Y.Y.); 2 Xinjiang Key Laboratory of Multilingual Information Technology, Xinjiang University, Urumqi 830017, China

**Keywords:** micro-expression recognition, attention mechanism, global feature, local feature, adaptive feature fusion, class imbalance

## Abstract

Micro-expressions are extremely subtle and short-lived facial muscle movements that often reveal an individual’s genuine emotions. However, micro-expression recognition (MER) remains highly challenging due to its short duration, low motion intensity, and the imbalanced distribution of training samples. To address these issues, this paper proposes a Global–Local Feature Fusion Network (GLFNet) to effectively extract discriminative features for MER. Specifically, GLFNet consists of three core modules: the Global Attention (LA) module, which captures subtle variations across the entire facial region; the Local Block (GB) module, which partitions the feature map into four non-overlapping regions to emphasize salient local movements while suppressing irrelevant information; and the Adaptive Feature Fusion (AFF) module, which employs an attention mechanism to dynamically adjust channel-wise weights for efficient global–local feature integration. In addition, a class-balanced loss function is introduced to replace the conventional cross-entropy loss, mitigating the common issue of class imbalance in micro-expression datasets. Extensive experiments are conducted on three benchmark databases, SMIC, CASME II, and SAMM, under two evaluation protocols. The experimental results demonstrate that under the Composite Database Evaluation protocol, GLFNet consistently outperforms existing state-of-the-art methods in overall performance. Specifically, the unweighted F1-scores on the Combined, SAMM, CASME II, and SMIC datasets are improved by 2.49%, 2.02%, 0.49%, and 4.67%, respectively, compared to the current best methods. These results strongly validate the effectiveness and superiority of the proposed global–local feature fusion strategy in micro-expression recognition tasks.

## 1. Introduction

Facial expressions (FEs) serve as a crucial medium for humans to convey emotional information and foster interpersonal relationships, typically manifesting as macro-expressions in daily life. However, when individuals attempt to conceal their true emotions under specific conditions, micro-expressions (MEs) emerge as critical cues to uncover their genuine feelings [1]. This attribute renders MEs highly applicable in a wide range of fields, including criminal interrogation, clinical diagnosis, and human–computer interaction [1,2,3]. Compared to macro expressions, MEs are characterized by subtle facial muscle movements and a very short duration (typically ranging from 1/25 to 1/5 of a second) [2,4], making them difficult for the human eye to observe and perceive. Consequently, the task of micro-expression recognition (MER) presents a significant challenge for humans and an even greater complexity for computers.

In recent years, the field of MER has made significant progress. However, due to the subtle variations inherent in MEs, extracting distinctive and discriminative features from these expressions remains a major research challenge [5]. Various feature description methods have been proposed, generally categorized into hand-crafted features and deep learning features [6]. Early works predominantly focused on hand-crafted feature extraction [7,8,9], but these traditional methods require manual parameter selection and algorithm design. Furthermore, different databases and tasks necessitate varying parameters and design choices, thereby increasing the complexity of these methods.

The success of end-to-end deep neural networks in various computer vision tasks has provided new insights for MER, with automatic learning methods such as convolutional neural networks (CNNs) gradually becoming mainstream in the field of MER. To more accurately capture subtle and discriminative features in MEs, researchers often first locate facial landmarks to identify regions of interest (ROIs), then crop these regions or apply masking to non-ROIs areas in order to extract local detail features [10,11,12]. However, such approaches often overlook two key issues: first, there may be overlapping regions between partitions, leading to redundant information and causing the model to overemphasize repeated features; second, while masking can highlight specific regions, it may also obscure context information relevant to expressions, thereby limiting the model’s ability to understand overall expression dynamics. These issues ultimately reduce the accuracy of MER. Unlike previous works, to avoid redundant information caused by overlapping regions, we design a simple yet effective Local Block (LB) module. The core idea of this module is to divide the feature map into non-overlapping local regions and decompose the deep residual network of ResNet into the corresponding number of branches. Each branch disregards the global background and focuses solely on a specific key facial region to extract features. By independently learning local discriminative features, the network’s sensitivity to fine-grained ME variations is enhanced, thereby improving recognition performance. Meanwhile, to prevent the model from overly concentrating on local details while neglecting crucial global features, we further design a Global Attention (GA) module. This module integrates a global attention mechanism into the basic residual block. By combining channel and spatial attention mechanisms, along with multilayer perceptrons and 3D convolutional structures, the GA module effectively reduces the dispersion of feature information, strengthens global interaction modeling, and ultimately improves the overall performance of the model.

In the field of MER, effective feature fusion strategies still require further exploration [12]. Previous studies [11,13,14] have mostly relied on simple operations such as feature concatenation, addition, or multiplication. However, these approaches struggle to fully exploit the complementarity and correlations between features, often leading to the loss of useful information or an increase in redundancy. To overcome this limitation, we propose an Adaptive Feature Fusion (AFF) module. This module first performs preliminary fusion of the contextual information from global features and the detailed representations from local features along the channel dimension. Then, an efficient channel attention mechanism is introduced to model the inter-channel dependencies of the fused features. In this way, a refined feature interaction mechanism and a dynamic weight allocation strategy are established, enabling the model to autonomously discover and enhance critical complementary information from both types of features.

Finally, to address the significant class imbalance in the datasets, we introduce the Class-Balanced Softmax Cross-Entropy (CB-SCE) loss to replace the conventional Cross-Entropy (CE) loss. Unlike CE loss, which assigns the same weight to all classes during training—resulting in better performance on majority classes while underperforming on minority classes—CB-SCE alleviates this issue by rebalancing the learning process across categories. Experimental results show that with the introduction of CB-SCE, the model achieves improvements of 2.05% in unweighted average recall (UAR) and 0.45% in unweighted F1-score (UF1).

In summary, the main contributions of this paper are as follows:

- We propose a new global–local feature fusion network called GLFNet. The network includes two branches: the GA module, designed to capture overall contextual information, ensuring the model can focus on important global features within the image; and the LB module, which divides the feature map into four sub-feature maps to more precisely capture local details and specific information.
- The AFF module is proposed, leveraging the complementarity and correlation between different features. By utilizing an attention mechanism for adaptive weight adjustment, this module enhances the expressive power of the features.
- The CB-SCE loss is introduced, which uses prior knowledge to weight the prediction scores of minority and majority class samples. This approach ensures the model pays more attention to categories that are rare and difficult to classify in the database.
- Extensive experiments conducted on multiple ME databases demonstrate that the proposed method achieves superior performance under both the Single Database Evaluation (SDE) and Composite Database Evaluation (CDE) protocols. In particular, under the SDE protocol, the proposed method outperforms existing approaches by 2.21% and 0.9% in terms of accuracy on the SAMM and CASME II datasets, respectively.

## 2. Related Work

MER typically involves two key steps: ME feature extraction and classification. Feature extraction is the core issue in MER [15]. Feature extraction methods can be broadly categorized into two types: traditional hand-crafted methods and deep learning methods.

### 2.1. Hand-Crafted Methods

Hand-crafted feature extraction methods were widely used in the early stages of MER. Common methods include Histogram of Oriented Gradients (HOG), Histogram of Oriented Optical Flow (HOOF), and Local Binary Pattern on Three Orthogonal Planes (LBP-TOP) for extracting ME features. HOG utilizes gradient information in local regions of the image and represents image features by calculating histograms of gradient directions at each pixel. Polikovsky et al. [8] adopted 3D HOG features to recognize movements in selected facial regions. This method subdivides facial expressions into specific regions and extracts 3D histograms for classification, effectively capturing subtle variations in facial expressions. Davison et al. [16] proposed a personalized micro-movement detection method based on 3D HOG. This method defines a facial template consisting of 26 regions based on the FACS and uses 3D HOG to extract temporal features for each region, providing a detailed description of facial motion changes. HOOF captures dynamic information of facial movements in MEs, where recognition largely depends on the motion vectors around the facial regions. Chaudhry et al. [17] proposed the Histogram of Oriented Optical Flow (HOOF) technique, which constructs histograms based on motion vectors. HOOF is scale-invariant and orientation-independent but is sensitive to illumination changes. Happy et al. [18] introduced the Fuzzy Histogram of Optical Flow Orientation (FHOFO) to overcome the limitations of HOOF. FHOFO uses histogram fuzzification to construct an appropriate angular histogram from the optical flow vectors, encoding temporal patterns robustly against changes in expression intensity. To address the issue of redundant information in complete ME sequences, Liong et al. [19] proposed the Bi-Weighted Oriented Optical Flow (Bi-WOOF) method, which computes optical flow between the onset and apex frames and encodes it into a histogram descriptor using local weighting based on magnitude and global weighting based on optical strain, effectively capturing the subtle motion patterns in MEs. LBP-TOP can simultaneously capture the spatial and temporal texture information in ME sequences. Yan et al. [20] employed it as a baseline method to evaluate the newly constructed CASME II dataset. Wang et al. [21] proposed LBP with Six Intersection Points (LBP-SIP) and LBP with Mean Orthogonal Planes (LBP-MOP). LBP-SIP and LBP-MOP improve feature extraction speed by reducing redundant information. Huang et al. [22] proposed the Effective Spatio-Temporal Completed Local Quantization Pattern (STCLQP) for MER, significantly enhancing facial ME analysis compared to LBP-TOP. Additionally, research has shown that color can also provide useful information for face recognition. Wang et al. [23] extracted LBP-TOP from Tensor-independent Color Space (TICS). To extract sparse information from ME sequences, Wang et al. [10] combined Robust Principal Component Analysis (RPCA) and Local Spatio-Temporal Directional Features (LSTD) to recognize MEs. RPCA extracts the fine motion information of MEs, while LSTD extracts the local texture features of the information.

Hand-crafted feature extraction methods rely on the expertise and prior knowledge of domain experts to design feature extraction algorithms based on prior knowledge of muscle movements and changes in facial regions [24]. The advantage of hand-crafted feature extraction methods lies in their ability to interpret the physical meaning of features and their lower computational resource requirements. However, these methods have certain limitations in terms of the effectiveness and generalization ability of the manually designed feature representations.

### 2.2. Deep Learning Methods

With the growth of ME databases and computational power, deep learning methods, particularly those based on CNNs, have also emerged. Xia et al. [25] proposed Spatio-Temporal Recurrent Convolutional Networks (STRCN) to establish relationships among facial position information and capture facial muscle contractions in different regions. This model combines multiple recurrent convolutional layers with a classification layer to extract visual features for facial emotion recognition. To reduce model complexity and computational costs, Liong et al. [6] introduced the Shallow Triple-Stream 3D CNN (STSTNet), which performs feature extraction by fusing three types of optical flow (i.e., optical strain, horizontal, and vertical optical flow fields), enabling the network to learn discriminative high-level and detailed features of MEs. Khor et al. [26] proposed a lightweight Dual-Stream Shallow Network (DSSN), which achieves MER by fusing heterogeneous input features, maintaining high performance while reducing model complexity. To better exploit local features, Chen et al. [27] proposed a novel Block-based Convolutional Neural Network (BDCNN) with implicit deep feature enhancement, where each image is divided into a set of small patches, followed by convolution and pooling operations on each patch. Li et al. [11] noted that different facial regions contribute unequally to MER and thus proposed a Local-to-Global Collaborative learning model (LGCcon). They extracted six ROIs using a sliding window with a stride of 1/6 of the face height and jointly learned emotional features from core local regions and global facial information. Nie et al. [28] proposed GEME, which integrates gender features as an auxiliary task into a multi-task learning framework for MER, and combines it with a class-balanced focal loss to effectively improve MER accuracy. Zhao et al. [29] were the first to exploit offset frames to complement motion details in MEs. They proposed a Channel Self-Attention Residual Network (CSARNet), which introduces a local feature augmentation strategy to highlight subtle facial muscle movements and builds a channel self-attention enhancement module to extract and refine features from motion flow images.

Graph Convolutional Network (GCN)-based approaches have also been applied in MER. Wei et al. [30] proposed SS-GN, the first to systematically explore the contribution of facial landmarks to MER. This network aggregates both low-order and high-order geometric motion information from facial landmarks, accurately capturing subtle geometric variations in MEs. The success of Vision Transformers (ViTs) in computer vision has also motivated researchers to extend them to MER. Hong et al. [31] proposed Later, which is the first work to apply Transformer to MER and effectively alleviates the data scarcity issue by incorporating optical flow motion features with a late fusion strategy. To overcome the limitations of CNNs in spatio-temporal feature extraction, Zhang et al. [32] proposed the SLSTT-LSTM network, which combines Transformer with LSTM and simultaneously models short- and long-term spatial and temporal dependencies through multi-head self-attention and temporal aggregation modules. Lei et al. [33] proposed AU-GCN, which simultaneously learns facial graph representations via a Transformer encoder and models Action Unit (AU) information with GCN as an adjacency matrix. Pan et al. [34] designed C3DBed, a 3D CNN embedded with a Transformer, combining the spatiotemporal feature extraction capability of 3D convolutions with the attention mechanism of Transformers. Bao et al. [35] proposed SRMCL, which innovatively combines supervised prototype-based memory contrastive learning with a self-expressive reconstruction auxiliary task, enhancing both the discriminability and generalization of features. Wang et al. [36] developed a Multi-scale Multi-modal Transformer Network (MMTNet), which integrates multi-scale feature learning with multi-modal representations from dynamic and optical flow features within a unified Transformer framework, thereby balancing fine-grained motion feature capture and efficient multimodal integration.

Although prior works [6,25] explored feature learning for MER, they failed to effectively capture subtle local features critical for MER. Chen et al. [27] recognized the importance of local features in extracting discriminative patterns but overlooked global contextual information. Li et al. [11] jointly considered global and local features but ignored the problem of redundant information caused by region partitioning, which reduced recognition accuracy. GCN-based methods [30,33] often rely on domain experts’ prior knowledge for defining facial regions, landmark selection, or AU configurations, which limits their adaptability. Existing ViT-based methods [35,36] suffer from two main drawbacks: on the one hand, their self-attention mechanism focuses more on learning global relationships and may overlook subtle local muscle movements essential for recognition; on the other hand, the complex structure of ViTs makes them less suitable for the limited-scale MER datasets, hindering their performance. Therefore, the objectives of this study are as follows: (1) To address the limited size of MER datasets, we propose GLFNet, which employs a lightweight backbone network to reduce the risk of overfitting; (2) to capture the subtle and weak-intensity features of MEs, we propose the LB module, which partitions input feature maps into non-overlapping blocks and applies independent convolutional operations to each block, thereby precisely extracting local subtle muscle movement information; (3) to compensate for CNN’s limitations in global feature modeling, we propose the GA module to learn the overall facial structure and contextual information; (4) to fully exploit the complementarity between local and global features, we propose the AFF module, which incorporates a dynamic weight allocation strategy to adaptively fuse the local detail features captured by the LB module with the global relational features learned by the GA module. Experimental results validate the effectiveness and superiority of the proposed method in MER tasks.

## 3. Proposed Method

**Figure 1:**
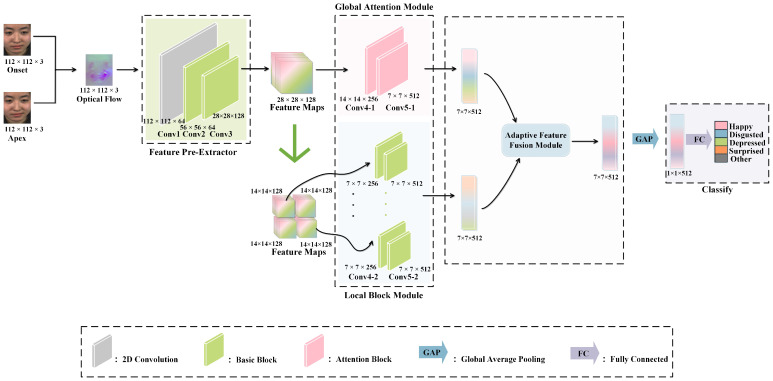
The structure of GLFNet. The proposed method comprises four components: feature pre-extraction, global attention module, local block module, and adaptive feature fusion module.

MEs are characterized by subtle motion amplitude, short duration, and feature concentration in key facial regions. Traditional single-feature modeling approaches struggle to simultaneously capture global facial dynamics and local fine-grained details [32,36]. To address this, we propose a dual-branch improved network, GLFNet, based on the ResNet [37] architecture. As illustrated in Figure 1, the network consists of four core components: a feature pre-extraction module, a GA module, a LB module, and a AFF module. Considering that ME datasets are typically small and exhibit imbalanced class distributions [2,38,39], we adopt the relatively shallow ResNet-10 as the backbone to reduce the risk of overfitting, which will be further validated in Section 4.5.1. Specifically, GLFNet first performs feature pre-extraction through the Conv1–Conv3 layers of the residual network to obtain mid-level facial features. The extracted feature maps are then fed into the dual-branch network for global and local feature learning. In the global branch, the GA module models the overall features to enhance the representation of global contextual information. In the local branch, the feature maps are partitioned along the spatial dimensions into several non-overlapping regions, and parallel CNN sub-networks independently learn the salient local features of each region. Finally, the global and local features are adaptively fused via the AFF module to fully exploit their complementary advantages. Additionally, to address the issue of class imbalance, we adopt the CB-SCE loss as the training objective, further improving the model’s generalization capability.

### 3.1. Preprocessing

Among the three most commonly used databases, CASME II [20], SAMM [40], and SMIC [41], the CASME II and SMIC databases have already provided cropped facial image video sequences. Since the SAMM database does not provide cropped images, we used the Dlib package to locate 68 facial landmarks and cropped the images based on the detected key points to reduce interference in the MER process. All cropped images were resized to 170×140.

MEs usually have very short durations and small amplitudes, making them difficult to recognize using traditional static image feature extraction methods. In the field of MER, the optical flow method is widely used to capture the subtle dynamic changes of MEs and extract effective temporal motion information. However, due to the low intensity of MEs, the optical flow changes between adjacent frames are weak and difficult to capture. To address this issue, we followed the method proposed by Liong et al. [19], using the TV-L1 [42] method to calculate the displacement between the onset frame and the apex frame to enhance the saliency of motion.

### 3.2. Global Attention Module

**Figure 2:**
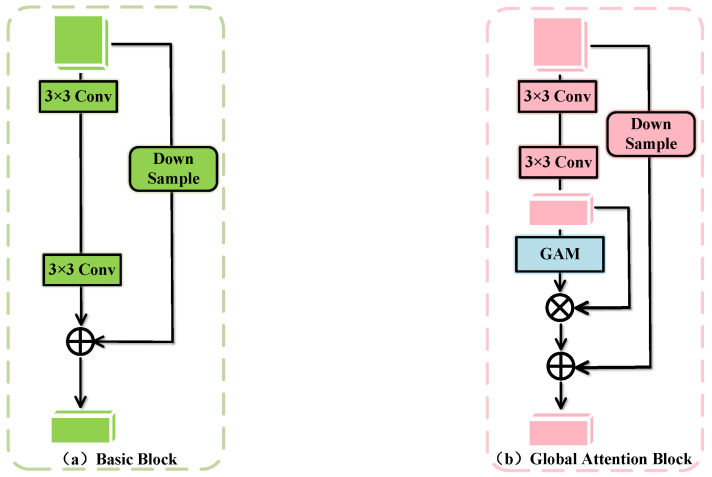
Two types of blocks used in GLFNet: (**a**) basic block, utilized for feature pre-extraction and local block module. (**b**) global attention block, employed for global attention module.

CNNs rely on the local receptive fields of convolutional kernels, which typically focus only on limited regions of the feature map, making it difficult to model long-range dependencies or global context. To address the limitations of ResNet in global feature extraction, we introduce a Global Attention Mechanism (GAM) [43] into its basic residual block, thereby constructing the GA module. The structure of the basic residual block in ResNet is shown in Figure 2a, and the architecture of the GA module is illustrated in Figure 2b. By combining the strengths of the Channel Attention Mechanism (CAM) and the Spatial Attention Mechanism (SAM), GAM captures salient features across three dimensions—channel, spatial width, and spatial height—thus mitigating the loss of critical information and the lack of cross-dimensional feature interaction caused by the convolution process in CNNs.

**Figure 3:**
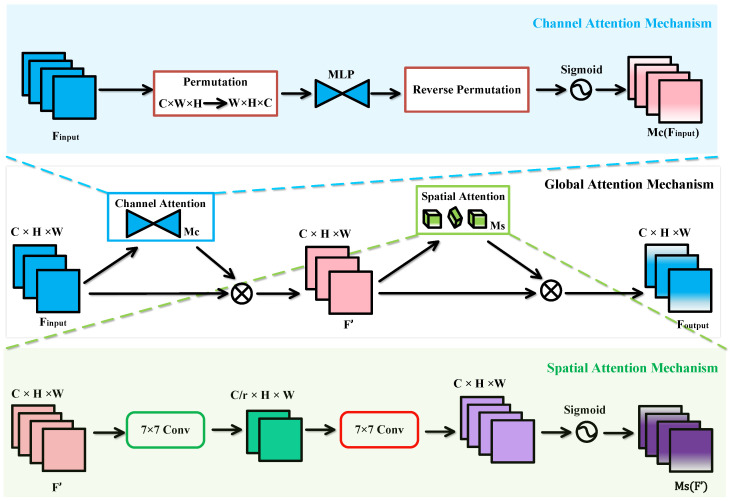
Schematic diagram of the GAM.

Figure 3 illustrates the implementation principle of the GAM, where input features pass through CAM and SAM sequentially, and are processed through a basic multiplication operation. The implementation formula of GAM is as follows:

(1) $$F^{\prime }=M_{c}\left(F_{input}\right)⊗F_{input}$$

(2) $$F_{output}=M_{S}\left(F^{\prime }\right)⊗F^{\prime }$$

where $F_{\mathrm{input}}$ represents the input features, $F_{\mathrm{output}}$ denotes the output features, $F^{\prime }$ is the intermediate feature, $M_{c}$ and $M_{s}$ are the CAM and SAM, respectively, and ⊗ signifies element-wise multiplication.

CAM performs dimensional transformation on the input feature map, employing a three-dimensional arrangement to preserve the original three-dimensional information. It then processes the transformed features with a Multilayer Perceptron (MLP) to enhance cross-dimensional correlations between channel and spatial representations, before converting them back to the original dimensionality. Finally, after applying a Sigmoid activation, the generated channel weights are used to reweight each channel of the original optical flow features, thereby strengthening the focus on the most informative channels.

SAM, similar to the Squeeze-and-Excitation (SE) attention mechanism [44], first reduces the number of channels and then increases it again. Specifically, SAM begins by applying a 7×7 convolutional kernel to the input to reduce the number of channels. It then uses another 7×7 convolutional kernel to convolve the result, restoring the number of channels to maintain consistency. By employing two successive 7 × 7 convolutional layers, SAM performs feature fusion, thereby enhancing the sensitivity of channel features to spatial information. Finally, after applying a Sigmoid function, the resulting spatial weights are used to adjust each position of the original optical flow features, emphasizing attention on crucial regions.

### 3.3. Local Block Module

To extract discriminative features, focusing on local regions is crucial. Previous methods typically involved locating ROIs through facial landmark points, cropping these regions, and segmenting the entire face into several smaller parts. However, this approach overlooks issues such as inaccuracies in landmark point localization, which can affect subsequent cropping and feature extraction, as well as the problem of information redundancy caused by overlapping cropped areas. Inspired by [45], the LB module specifically focuses on four local regions, disregarding the entire facial area. By ignoring the full face region, the model can concentrate more on the core expression areas of the face, reducing noise and interference from non-essential regions. The LB module decomposes Conv4 and Conv5 into four parallel branches, each corresponding to one part. This decomposition structure allows each branch to independently learn and extract features relevant to its specific part, thereby producing more discriminative and enriched feature representations for each segment. Consequently, the network becomes more sensitive to subtle facial movements and expression changes, enhancing its ability to perceive fine-grained features.

**Figure 4:**
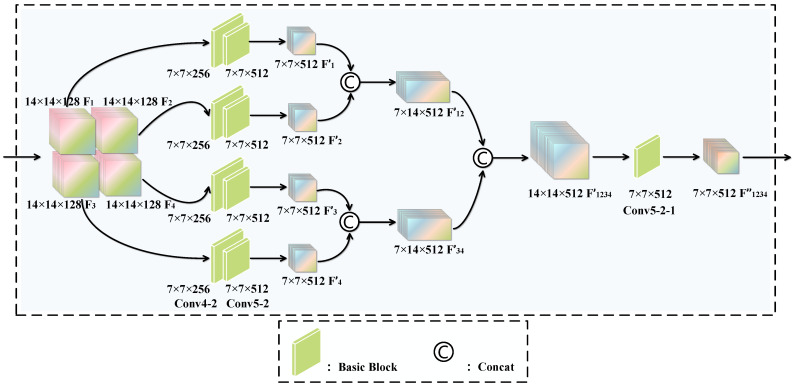
Schematic diagram of the LB module.

As illustrated in Figure 4, our method divides the intermediate feature map into several non-overlapping local feature maps. Through convolution operations, these local feature maps can autonomously focus on and capture local salient features. Specifically, after the feature pre-extraction module, we partition the feature map $M\in R^{28\times 28\times 128}$ (where 28 is the spatial size and 128 is the number of channels) along the spatial axis into four non-overlapping local feature maps $M_{i}\in R^{14\times 14\times 128}$, where i∈{1,2,3,4}, to better match the typical locations where MEs occur. It is noteworthy that each local feature map $M_{i}$ retains the same number of channels as the original feature map *M*, but its spatial dimensions are halved.

In the LB module, the four 14×14×128 feature maps are processed independently by their corresponding branch networks to produce four 7×7×512 feature maps. These four feature maps are then concatenated along the spatial axis. Specifically, the processed feature maps from the top-left and top-right regions are concatenated along the third dimension to form a feature map representing the upper half of the face. Similarly, the processed feature maps from the bottom-left and bottom-right regions are concatenated along the third dimension to form a feature map representing the lower half of the face. Next, the concatenated upper and lower feature maps are combined along the second dimension to produce a feature map of size 14×14×512.

Finally, a basic residual network block is added, which includes a convolutional layer with 512 input channels and 512 output channels, with a stride of 2. This final feature map is processed through this convolutional layer to yield a new feature map of size 7×7×512. By adding this convolutional layer, we reduce the spatial dimensions of the feature map while preserving important information, thereby facilitating efficient integration with the extracted global features.

### 3.4. Adaptive Feature Fusion Module

**Figure 5:**
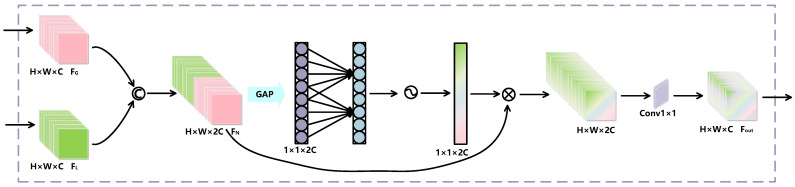
Schematic diagram of the AFF module.

Due to the varying contributions of global and local features in MER tasks, simply adding or concatenating these two types of features can lead to information overlap or redundancy. To better utilize global and local features, we designed the AFF module, incorporating the Efficient Channel Attention (ECA) mechanism [46], which is a lightweight channel attention approach to enhance feature representation capability. The specific structure is illustrated in Figure 5.

Firstly, the input data consists of global features $F_{G}\in R^{7\times 7\times 512}$ and local features $F_{L}\in R^{7\times 7\times 512}$. These features are concatenated along the channel dimension to form a new feature map $F_{N}\in R^{7\times 7\times 1024}$. Then, $F_{N}$ is processed through the ECA module. The principle of the ECA attention mechanism is to perform local cross-channel interaction using one-dimensional convolution, avoiding information loss caused by dimensionality reduction and fully leveraging spatial and temporal channel information. The specific implementation steps are as follows:

Firstly, global average pooling is applied to each channel of the feature map $F_{N}$ to generate a vector describing the channel features, as shown below:

(3) $$F_{N1}=\mathrm{GAP}\left(F_{N_{c}}\right)$$

where GAP(·) denotes global average pooling, $F_{N_{c}}$ is the feature map $F_{N}$ of the *c*-th channel, and $F_{N1}$ is the pooled feature vector.

Then, a one-dimensional convolution is applied to the global average pooled vector to learn channel relationships and weights, and the weights ω for each channel are obtained using the Sigmoid activation function, calculated as follows:

(4) $$\omega =\sigma ({\mathrm{Conv}}_{1D}\left(F_{N1}\right))$$

where σ(·) denotes the Sigmoid activation function, and ${\mathrm{Conv}}_{1D}\left(\cdot \right)$ represents the one-dimensional convolution operation. The size *k* of the convolution kernel is adaptive and determined by the number of channels *C*, calculated as:

(5) $$k={\left|\frac{{log}_{2}\left(C\right)}{\gamma }+\frac{b}{\gamma }\right|}_{odd}$$

where ${\left|t\right|}_{\mathrm{odd}}$ denotes the nearest odd number to t, and γ and *b* are adjustment parameters, which we set to 2 and 1, respectively.

The channel attention weights are used to reweight the original features, enhancing the weights of useful channels and reducing the weights of irrelevant channels to more effectively capture important information in MEs. Finally, to improve the model’s computational efficiency, we perform a 1×1 convolutional dimensionality reduction on the feature map processed by the ECA mechanism, obtaining the final feature $F_{out}\in R^{7\times 7\times 512}$. By applying the 1×1 convolution, we can reduce the number of channels in the feature tensor while maintaining model performance, thereby decreasing the model’s parameter count and computational complexity.

By incorporating the ECA mechanism, we mitigate feature redundancy and information overlap, thereby enhancing the utilization of the disparities and complementarities between global and local features. This improvement leads to a more effective and accurate performance in MER.

### 3.5. Loss Function

Given the difficulty in collecting MEs, especially for emotions that are hard to evoke and capture, such as ‘Happiness’ and ‘Fear’ [24], data imbalance is further exacerbated. Typically, for imbalanced databases, models may tend to learn more from classes with a larger number of samples while ignoring those with fewer samples. To address this issue, an effective imbalance loss strategy is needed; therefore, we introduce Class-Balanced Softmax Cross-Entropy (CB-SCE) loss [47].

Class-Balanced (CB) loss adjusts the loss function by introducing a weighting factor to balance the contribution of different class samples. This weighting factor is inversely proportional to the effective number of samples, meaning classes with fewer samples have higher weights, while classes with more samples have lower weights. This strategy of adjusting the loss function helps improve the model’s ability to learn from minority classes and enhances classification performance on imbalanced databases. By balancing the contribution of different class samples, CB Loss can improve the model’s generalization ability and overall performance. The CB Loss can be expressed as:

(6) $$L_{\mathrm{CB}}\left(p,y\right)=\frac{1}{E_{n_{y}}}L\left(p,y\right)=\frac{1-\beta }{1-\beta^{n_{y}}}L\left(p,y\right)$$

where y∈{1,2,…,C} is the label of the input sample *x*, *C* is the total number of classes, $p={\left[p_{1},p_{2},\ldots ,p_{C}\right]}^{⊤}$ is the model’s probability estimate for each class, and $n_{y}$ is the number of samples in the ground truth class *y*. The weighting factor is $\frac{1-\beta }{1-\beta^{n_{y}}}$, with the hyperparameter β∈[0,1). Notably, β=0 corresponds to no reweighting, while β→1 corresponds to reweighting by the inverse class frequency.

CB-SCE Loss is an improvement over Cross-Entropy (CE) loss, employing a binary CE loss function with class weights. This loss function calculates the loss value based on the predicted probability distribution and the one-hot encoded true labels, using dynamic weights for each class to address class imbalance. This improvement effectively mitigates the performance degradation caused by data imbalance, enhancing the accuracy and stability of the model in practical applications. The softmax function assumes that each class is mutually exclusive and calculates the probability distribution for each class by exponentiating and normalizing the inputs. Specifically, for each class ∀i∈{1,2,…,C}, the probability distribution of all classes is given by $p_{i}=\frac{\exp(z_{i})}{\sum_{j=1}^{C}\exp\left(z_{j}\right)}$. Therefore, the Softmax Cross-Entropy (SCE) loss and CB-SCE Loss are written as follows:

(7) $$L_{\mathrm{SCE}}\left(z,y\right)=-\log\left(\frac{\exp\left(z_{y}\right)}{\sum_{j=1}^{C}\exp\left(z_{j}\right)}\right)$$

(8) $$L_{\mathrm{CB}-\mathrm{SCE}}\left(z,y\right)=-\frac{1-\beta }{1-\beta^{n_{y}}}\log\left(\frac{\exp\left(z_{y}\right)}{\sum_{j=1}^{C}\exp\left(z_{j}\right)}\right)$$

where $z={\left[z_{1},z_{2},\ldots ,z_{C}\right]}^{⊤}$ represents the model’s output predictions for all classes, *C* is the total number of classes, and the weighting factor is $\frac{1-\beta }{1-\beta^{n_{y}}}$, with the hyperparameter β∈[0,1).

## 4. Experiments

To validate the effectiveness of GLFNet, we conducted extensive experiments on three popular ME databases: CASME II [20], SAMM [40], and SMIC [41]. In this section, we will describe the experimental details, including the databases used, experimental settings, results, and ablation studies.

### 4.1. Databases

SMIC [20] was recorded at 100 fps with a resolution of 640×480. The sample labels include three emotion types: ‘Positive’, ‘Negative’, and ‘Surprise’ with MEs annotated using a three-class classification scheme. Emotion labels are based on self-reports from participants, which may be inaccurate, and vertex frames and AUs labels are not provided.

CASME II [40] was recorded with a high-speed camera at 200 fps and a face resolution of 280×340. The participants, with an average age of 22.59 years, were all Asian. Sample labels encompass five different emotion types, including vertex frames, AUs, and emotions. MEs are annotated using a five-class classification scheme: ‘Happiness’, ‘Disgust’, ‘Suppression’, ‘Surprise’, and ‘Others’.

SAMM [41] also employed a high-speed camera at 200 fps with a face resolution of 400×400. The participants had an average age of 33.24 years and came from 13 different ethnicities. Sample labels include eight emotion types: ‘Happiness’, ‘Fear’, ‘Surprise’, ‘Anger’, ‘Disgust’, ‘Sadness’, ‘Contempt’, and ‘Others’. All samples are labeled with emotions, vertex frames, and AUs.

All three databases were recorded in fully controlled laboratory environments with lighting conditions managed to prevent image flicker. In addition to these databases, we use the combined database proposed by MEG2019 to validate our algorithm’s performance [48]. This database integrates all ME samples from the aforementioned three databases into a single composite database. Due to significant annotation differences among the three databases, the combined database standardizes the emotion labels across all three sources. The emotion labels are re-annotated as ‘Positive’, ‘Negative’, and ‘Surprise’. The sample distribution for the three databases and the combined database is shown in Table 1.

### 4.2. Evaluation Metrics

To demonstrate the effectiveness of SRMCL, we employed two popular evaluation standards: Sole Database Evaluation (SDE) and Composite Database Evaluation (CDE). In SDE, common evaluation metrics are Accuracy (ACC) and F1-Score (F1). Typically, ACC measures the proportion of correct predictions out of the total evaluated samples. However, accuracy can be sensitive to imbalanced data. To address this issue, the F1 takes into account True Positives (TP), False Positives (FP), and False Negatives (FN), thus revealing the true classification performance.

(9) $$Acc_{c}=\frac{TP_{c}}{N_{c}}$$

(10) $$F1_{c}=\frac{2TP_{c}}{2TP_{c}+FP_{c}+FN_{c}}$$

where $TP_{c}$, $FP_{c}$, and $FN_{c}$ represent the true positives, false positives, and false negatives for each emotion class *c*, respectively; $N_{c}$ denotes the number of samples for each emotion class *c*; $ACC_{c}$ and $F1_{c}$ represent the ACC and F1 for each class, respectively.

For CDE, which combines multiple databases resulting in significant class imbalance, the performance is evaluated using Unweighted F1 score (UF1) and Unweighted Average Recall (UAR). UF1, also known as macro-average F1, is calculated by averaging the F1 scores of each class. In an imbalanced multi-class setting, UF1 provides equal emphasis on rare and common classes. UAR is defined as the average accuracy across each class divided by the number of classes, without considering the number of samples per class. UAR helps to mitigate bias introduced by class imbalance, thus it is referred to as balanced accuracy.

(11) $$UF1=\frac{1}{C}\sum_{c}F1_{c}$$

(12) $$UAR=\frac{1}{C}\sum_{c}Acc_{c}$$

where *C* denotes the number of emotion classes.

### 4.3. Experimental Setup

To ensure that the input images meet specific requirements, we performed preprocessing operations including normalization and resizing. To enhance the diversity of the training data, we applied data augmentation techniques such as random cropping and random horizontal flipping. The images were ultimately resized to 112×112 pixels. We adopted ResNet-10 as the backbone network architecture. All experiments were conducted on Ubuntu 20.04.6 LTS, using PyTorch 1.10.0 and Python 3.9.18, and the model was implemented and executed on a GeForce RTX 2080 Ti GPU. For model optimization, we used the Adam optimizer with a weight decay parameter set to $1\times 10^{-4}$. Additionally, we set the batch size to 8, the initial learning rate to $1\times 10^{-4}$, and the number of epochs to 80, utilizing Leave-One-Subject-Out (LOSO) cross-validation [49] to evaluate and report the MER performance.

As shown in Table 1, the datasets used in this study exhibit a clear class imbalance. For example, in the CASME II dataset, the “Others” category accounts for more than 40% of the samples, while the “Surprise” category comprises only 10.2%. The imbalance is even more pronounced in the SAMM dataset, where the “Contempt” category accounts for just 8.1%, whereas the “Angry” category reaches 41.4%. Such distribution disparities can easily bias the model toward majority classes during training, thereby weakening its ability to recognize minority classes. To mitigate this issue, we replace the standard CE loss with the CB-SCE loss, enhancing the model’s learning capability for minority and hard-to-classify samples.

### 4.4. Experimental Results

#### 4.4.1. Experimental Results Under SDE

We evaluated the performance of the proposed method under the SDE standard and compared it with previous handcrafted and deep learning methods. The results demonstrate that our method excels in the MER task, significantly outperforming traditional hand-crafted methods and most existing deep learning methods. The specific results are shown in Table 2.

Compared with the state-of-the-art CNN-based method CSARNet [29], our approach achieves superior performance across all datasets. In the five-class classification task on the CASME II database, our method improves ACC by 0.9% and F1 by 1.13% compared to the current state-of-the-art method SRMCL [35], with both metrics exceeding 83%. In the five-class classification task on the SAMM database, our method also achieved optimal performance, with GLFNet leading by 3.31% in ACC and 6.79% in F1 compared to SRMCL. Even when compared to the current highest recognition accuracy method C3D-Bed [34], our method surpassed it by 2.21% in ACC and 0.62% in F1. However, GLFNet slightly underperformed compared to SS-GN [30] in F1. We attribute this to the fact that SS-GN employs a graph network structure and introduces an adaptive AU loss method, dynamically adjusting the model’s attention to different AUs during training, which is highly effective for ME modeling on small databases. Nevertheless, GLFNet significantly outperformed SS-GN on the larger CASME II database. Although GLFNet performs relatively poorly on the SMIC dataset, its overall performance still surpasses most existing methods. Its ACC is only 0.90% lower than the current best-performing SRMCL, and its performance is comparable to MMTNet, with only a slight disadvantage in F1 score. Upon analysis, we attribute this to the relatively low image quality in the SMIC dataset, which likely contains more noise. SRMCL incorporates a Self-Expression Reconstruction mechanism that reconstructs the input by randomly dropping patches, thereby enhancing model robustness. Meanwhile, MMTNet leverages both optical flow and dynamic features as input, where dynamic features compensate for temporal information and help mitigate the impact of noise to some extent. However, on higher-quality datasets such as CASME II and SAMM, GLFNet clearly outperforms both SRMCL and MMTNet. Based on these results, we suggest that future work should further focus on enhancement and adaptation strategies for low-quality images to improve model robustness in diverse data environments.

The outstanding performance of GLFNet on multiple commonly used databases further validates the effectiveness of combining global and local features. The complementary nature of global and local features significantly enhances the model’s discriminative ability. We hypothesize that our method can exhibit even better performance on larger databases, which will be verified in experiments under the CDE standard.

**Figure 6:**
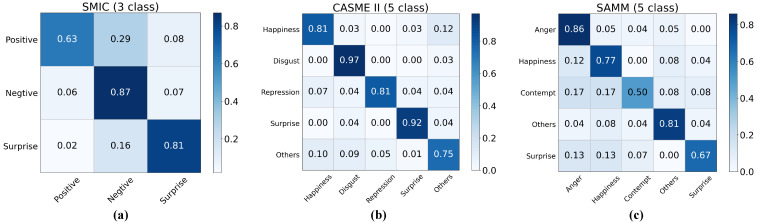
Confusion matrix of classification results under the SDE standard: (**a**) confusion matrix of the three-class classification results for the SMIC database, (**b**) confusion matrix of the five-class classification results for the CASME II database, (**c**) confusion matrix of the five-class classification results for the SAMM database.

Figure 6 shows the confusion matrix of the classification results under the SDE standard. According to the confusion matrix of the five-class classification results from the SAMM database, it is evident that GLFNet performs poorly on the ‘Contempt’ and ‘Surprise’ categories. This may be due to the relatively small number of samples for these two categories in the SAMM database, accounting for only 8% and 11% of the total samples, respectively. Due to insufficient samples, the model may lack adequate data to effectively learn and distinguish the features of these categories, resulting in decreased classification performance for these categories.

#### 4.4.2. Experimental Results Under CDE

To further validate the effectiveness and generalization ability of the proposed method, we conducted three-class experiments under the CDE standard, achieving state-of-the-art results across all databases, as shown in Table 3.

Specifically, compared to the current best method, SRMCL, GLFNet’s UF1 and UAR on the CASME II database were higher by 0.49% and 1.04%, respectively; on the SAMM database, they were higher by 4.67% and 0.65%, respectively; and on the SMIC database, GLFNet’s UF1 was higher by 2.02%, although UAR was slightly lower by 0.24%. These results are consistent with the experiments under the SDE standard, confirming our previous conjecture that SRMCL’s strategy of randomly discarding patches to reconstruct inputs may perform better on lower-quality databases, but is less effective than our method on higher-quality databases. Notably, GLFNet significantly outperformed other methods that emphasize the importance of local features (such as AU-GCN [33], BDCNN [27], FRL-DGT [12], etc.). This superior performance is attributed to GLFNet’s emphasis on both local and global features, effectively enhancing MER performance by efficiently integrating local and global features.

**Figure 7:**
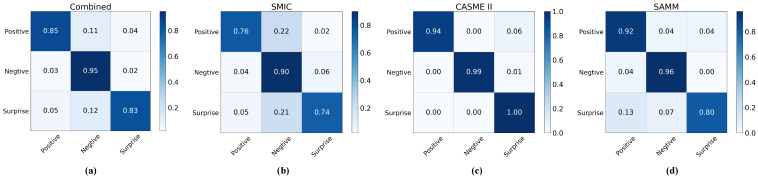
Confusion matrices of classification results under the CDE criterion: (**a**) confusion matrix of the composite database for the three-class classification, (**b**) confusion matrix of the SMIC database for the three-class classification, (**c**) confusion matrix of the CASME II database for the three-class classification, (**d**) confusion matrix of the SAMM database for the three-class classification.

Based on the confusion matrices of classification results under the CDE standard shown in Figure 7, we can observe that GLFNet achieved remarkable results on the composite database. The ‘Negative’, ‘Positive’, and ‘Surprise’ categories attained accuracies of 0.95, 0.85, and 0.83, respectively. Notably, the ‘Negative’ category achieved the highest recognition results across all databases. This may be attributed to the larger number of training samples for the ‘Negative’ category in these databases, enabling the model to more thoroughly learn and understand the features of this category.

Compared to the performance on the CASME II and SAMM databases, the lower performance of GLFNet on the SMIC database can be primarily attributed to the difference in frame rates used for capturing samples. The SMIC database employs a frame rate of 100 fps, while the other two databases use a frame rate of 200 fps. The lower frame rate limits the accurate identification of apex frames in the SMIC database. Additionally, the images in the SMIC database contain background noise such as shadows, highlights, lighting variations, and flickering lights caused by the experimental setup of the database.

#### 4.4.3. Results Discussion

In experiments conducted on three datasets under two mainstream MER evaluation protocols, the proposed GLFNet achieved state-of-the-art performance. It is worth noting, however, that under the SDE protocol, SFML-Net performs worse than SRMCL on the SMIC dataset. We attribute this primarily to the characteristics of SMIC, which was collected in 2013; due to the hardware limitations at that time, the camera frame rate was low and the original video resolution was limited. SRMCL significantly improves its handling of low-quality data by introducing a Self-Expression Reconstruction mechanism, which reconstructs the input through randomly dropping image patches. This approach enables SRMCL to achieve excellent performance on SMIC. Inspired by this, our future work will focus on exploring low-quality data enhancement and reconstruction techniques, aiming to improve the model’s stability and adaptability when handling noisy or low-resolution images.

On the SAMM dataset, although GLFNet outperforms existing state-of-the-art methods under both protocols, the confusion matrices in Figure 6 reveal that the model still struggles with the “Contempt” and “Surprise” categories. Beyond the limited training samples, we believe this performance bottleneck is partly due to insufficient global feature modeling capability. Despite introducing the GAM in GLFNet to mitigate CNNs’ limitations in capturing long-range dependencies, the model still exhibits shortcomings in capturing global semantic relationships across local regions. In our subsequent work, we explore hybrid CNN-ViT architectures to leverage CNNs’ strengths in local feature extraction alongside ViT’s capability for modeling global dependencies, aiming to further improve performance in MER.

Furthermore, the current model uses only optical flow features as input, which limits the completeness of motion information. Future work could explore additional feature types; for example, MMTNet incorporates dynamic features into MER to complement the temporal motion information not captured by optical flow, thereby further enhancing model performance in MER.

### 4.5. Ablation Study

To validate the effectiveness of each component in the GLFNet model, we conducted a series of ablation experiments on the CASME II dataset. In these experiments, we separately examined the impact of the GA module, LB module, AFF module, and CB-SCE. The experimental results are presented in Table 4.

First, when only the GA module is introduced, the model achieves 79.92% ACC, 0.7783 UF1, and 0.7624 UAR. The GA module leverages the global attention mechanism by integrating channel and spatial attention, together with multilayer perceptrons and 3D convolutional structures, effectively reducing feature dispersion and improving the backbone model’s limitations in global feature extraction. When only the LB module is added, the model achieves an ACC of 81.17%, which is 1.25% higher than that of the GA-only setting. The LB module focuses on local details of the image, capturing subtle regional features through partitioning the feature map, which enables the model to concentrate on fine-grained local variations and extract more discriminative features. When both GA and LB are enabled, the performance improves, with ACC reaching 81.59%. This indicates that the initial fusion of global contextual information and local details allows the features to complement each other, thereby overcoming the limitations of using a single branch. With the additional inclusion of the AFF module, the model’s ACC significantly increases to 84.10%, indicating that the adaptive feature fusion mechanism can more effectively integrate global and local information, yielding more comprehensive, precise, and discriminative feature representations. Finally, when both AFF and CB-SCE are incorporated into the complete model, GLFNet achieves the best performance across all metrics. These results clearly demonstrate that the proposed modules are complementary in MER and that their joint utilization significantly enhances the discriminative power of the model.

#### 4.5.1. Influence of Backbone Networks

To investigate the impact of the backbone network on GLFNet, we conducted a set of ablation experiments, in which the model was trained and evaluated using the CE loss by default. The experimental results have been summarized and are presented in Table 5.

As shown in Table 5, the recognition performance of ResNets with different depths on the CASME II dataset exhibits certain variations. Overall, the performance does not show a strictly increasing trend with greater network depth. ResNet-10 achieves the best results in terms of ACC (76.15%) and F1 (0.7549), while ResNet-18 obtains the highest scores in UF1 (0.7445) and UAR (0.7453). This indicates that deeper networks do not necessarily provide clear advantages in MER; on the contrary, they may suffer from overfitting due to the limited dataset size, which negatively impacts overall performance. Considering the balance across all four metrics, both ResNet-10 and ResNet-18 demonstrate strong feature extraction capabilities, with ResNet-10 showing superior performance in overall accuracy. Therefore, ResNet-10 is adopted as the backbone network in subsequent experiments.

In addition, considering that the overall performance gap between ResNet-10 and ResNet-18 is relatively small, we conducted a set of comparative experiments to further examine the impact of different backbones on the model. As shown in Table 6, although the single GA module and GB module based on ResNet-18 improve recognition accuracy to some extent, their overall performance still falls short of the ResNet-10 implementation. This is mainly due to the limited scale of ME datasets, where a larger number of model parameters tends to cause overfitting. Nevertheless, the results of GLFNet based on ResNet-18 are consistent with those of ResNet-10 in terms of overall trends, confirming that the individual branch modules can effectively enhance the backbone model’s performance. It is worth noting that the LB module achieves a greater improvement in recognition accuracy than the GA module, indicating that the LB module possesses stronger discriminative power in local feature extraction and is more effective in modeling subtle ME variations.

#### 4.5.2. Influence of Feature Fusion

We realized that simple operations such as feature Concatenation (Concat), Addition (Add), or Multiplication (Mul) may not fully exploit the complementary information from the two branches. Therefore, we designed a set of comparative experiments to investigate the impact of different feature fusion strategies on model performance. During the experiments, all other components of the model were kept unchanged, and the loss function was fixed as CB-SCE loss to ensure fairness and comparability of the results. The experimental results are presented in Table 7.

From the results, it can be observed that traditional simple fusion methods exhibit certain limitations. Specifically, the Add operation performs relatively better among the metrics, achieving an ACC of 83.26%, outperforming both concatenation and multiplication. However, although Add can partially preserve the complementary information between features, it remains insufficient in addressing feature redundancy and differentiated weight allocation. Concat increases the feature dimensionality, but as it merely relies on simple stacking, it fails to emphasize discriminative information. Mul performs the worst across all metrics, indicating that it tends to cause information loss during the fusion process and struggles to effectively model the relationships between different features. In contrast, our proposed AFF module achieves the best performance across all metrics, with ACC improvements of 0.84% over Add and 1.67% over Concat. These results demonstrate that AFF, by introducing an adaptive weight learning mechanism, can more effectively integrate global and local features, overcoming the shortcomings of traditional methods in information utilization, and is better suited for capturing the subtle and transient dynamics of MEs.

#### 4.5.3. Influence of Loss

To validate the effectiveness of the CB-SCE loss function, we conducted a series of experiments comparing it with the standard CE loss and the commonly used Focal Loss [50]. Focal Loss is an improved loss function based on cross-entropy, designed to alleviate class imbalance and enhance the learning of hard-to-classify samples. Given its wide application and representativeness in related tasks, we included it as one of the comparison backbones in our experiments. The results are summarized in Table 8. It is noteworthy that, in cases of class imbalance, UAR focuses on the recall rate for each class and directly reflects the model’s performance in handling imbalanced data. UF1 provides a comprehensive evaluation for each class. In this set of experiments, we mainly focused on these two metrics.

Compared to CE loss and FL, CB-SCE loss shows significant improvements in handling class imbalance. Specifically, CB-SCE loss improves UAR and UF1 by 2.05% and 0.45% over CE loss, respectively, and by 0.72% and 0.38% over FL. FL, commonly used to address class imbalance, improves UAR and UF1 by 1.33% and 0.05% compared to CE loss. Despite FL’s improvements in dealing with class imbalance, its effectiveness is still less than that of CB-SCE loss, and this improvement comes at the cost of reduced ACC. Further analysis suggests that FL’s focal mechanism may lack flexibility in precisely adjusting the loss weights for each class. In contrast, CB-SCE loss dynamically adjusts weights based on the sample size of each class, making it more effective in addressing class imbalance challenges in the ME database.

**Figure 8:**
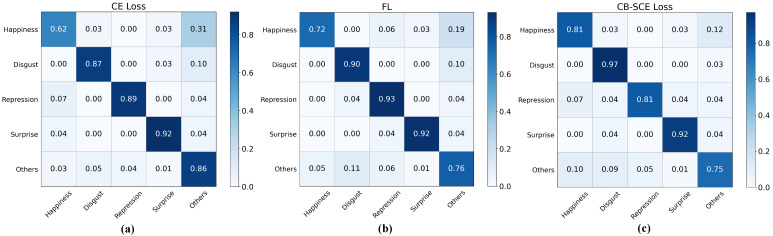
Confusion matrices for different loss functions: (**a**) confusion matrix for five-class classification with cross-entropy loss, (**b**) confusion matrix for five-class classification with focal loss, (**c**) confusion matrix for five-class classification with class-balanced softmax cross-entropy loss.

To visually demonstrate CB-SCE loss’s effectiveness in alleviating class imbalance issues, we have plotted confusion matrices under different loss functions, as shown in Figure 8. Observing these matrices, we can clearly see that both FL and CB-SCE loss improve the accuracy of the ‘Happiness’ and ‘Disgust’ categories, which had relatively low accuracy with CE loss, with CB-SCE loss showing a more significant enhancement. Although the accuracy of the ‘Others’ category was also relatively low with CE loss, both FL and CB-SCE loss show a decline in accuracy for this category. This phenomenon may be attributed to the fact that the “others” category constitutes 40% of the total sample size, meaning that this category has a relatively larger number of samples. FL and CB-SCE loss have a more noticeable effect on categories with fewer samples, thus their performance on larger categories may not meet expectations. These results indicate that CB-SCE loss outperforms FL and CE loss in addressing class imbalance issues, especially in significantly improving the classification performance of minority classes.

### 4.6. Complexity Analysis

We compared the computational complexity of the proposed GLFNet with several state-of-the-art methods in terms of parameter count, runtime, and memory consumption; the detailed results are presented in Table 9. The experiments show that GLFNet has 25.82 M parameters, a runtime of 81.67 s, and a memory footprint of 98.30 MB. Although GLFNet’s complexity is higher than that of earlier methods such as STSTNet, DSSN, and BDCNN, it requires substantially fewer parameters and less runtime compared to more recent approaches including SLSTT-LSTM, HTNet, and SRMCL, indicating that GLFNet achieves competitive performance while offering improved computational efficiency.

Early CNN-based methods, such as STSTNet, have a very small number of parameters (only 0.00167 M); however, due to their shallow network structures, they struggle to adequately model the complex features of facial MEs, resulting in limited performance on challenging tasks. ViT-based models, such as HTNet and SRMCL, improve the ability to capture long-range dependencies through self-attention mechanisms, but at the cost of substantial computational overhead, with parameter counts reaching 149.57 M and 342.15 M, respectively, and longer execution times, which restricts their applicability in resource-constrained scenarios. Hybrid architectures like SLSTT-LSTM attempt to combine ViT and LSTM to leverage spatiotemporal feature modeling, yet their parameter count of 93.05 M still falls short of meeting lightweight requirements.

In contrast, the proposed GLFNet achieves an effective balance between model complexity and computational efficiency while maintaining strong feature representation capability. With only 36.97 M parameters, it is smaller than mainstream ViT models and has a shorter execution time. Although its parameter count is higher than early lightweight CNN models, experimental results in Table 2 and Table 3 demonstrate that GLFNet significantly outperforms methods such as STSTNet and BDCNN across all datasets. Compared with recently proposed pure ViT models and other hybrid architectures, GLFNet not only exhibits higher runtime efficiency in most experimental scenarios but also achieves superior recognition performance.

### 4.7. Visualization

**Figure 9:**
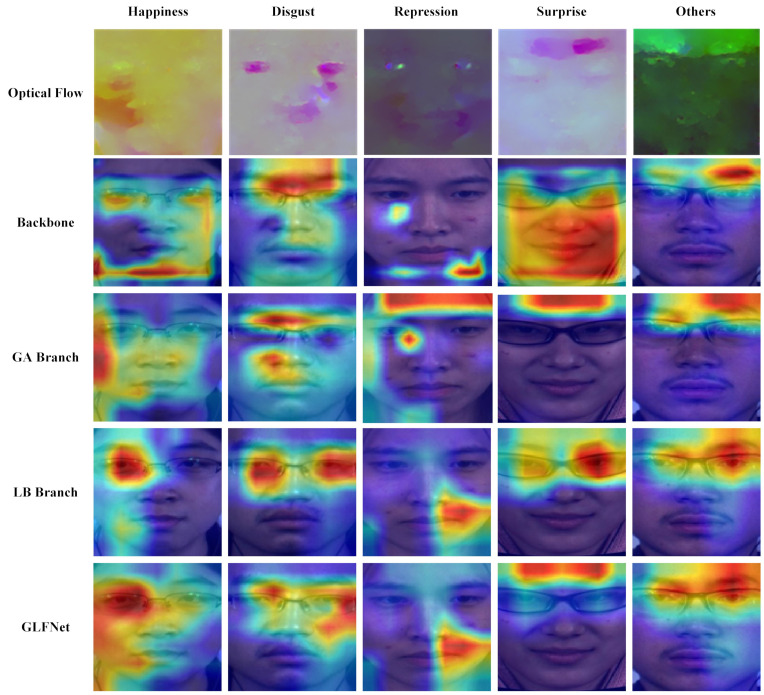
The Grad-CAM visualization results for the backbone, GA branch, LB branch, and GLFNet.

To verify the model’s ability to recognize specific emotions and determine whether highly activated regions correspond to specific motion locations, we visualized the backbone, GA, LB, and GLFNet models. The results of the Gradient-weighted Class Activation Mapping (Grad-CAM) [51] visualizations are presented in Figure 9.

To validate the effectiveness of our proposed GA module in extracting global features, we visualized the attention maps generated by the GA branch. By comparing these maps with backbone results and optical flow features, we observed that for emotions like ‘Surprise’ and ‘Disgust’, where muscle movement locations show minor differences, the GA branch successfully achieved more precise localization of motion regions compared to the backbone model. Furthermore, when dealing with emotions with significant differences in muscle movement areas (such as the eyebrows and mouth for ‘Happiness’), the model accurately focused on key motion areas. This confirms the effectiveness of the GA module in global feature extraction.

To validate the effectiveness of our proposed LB module in tracking subtle facial muscle movements, we visualized the feature maps generated by the LB branch. The visualization results clearly show that the feature maps produced by the LB module are very helpful in accurately localizing facial muscle movements. Notably, in ‘Surprise’ samples, the LB module mainly focused on the upper eyebrow region, while in ‘Happiness’ samples, the attention was more on the eyes and corners of the mouth. These findings further verify the LB module’s sensitivity in capturing subtle motion features in different emotional expressions.

Finally, we visualized the feature maps generated by GLFNet. The results confirmed the effectiveness of our previous work, as the visualization results of GLFNet successfully integrated the characteristics of both the GA and LB branches. GLFNet accurately captured motion features in different emotional expressions and localized muscle movement regions with higher precision.

## 5. Conclusions

In this paper, we propose a Global–Local Feature Fusion Network (GLFNet), designed to extract both global features and local features from four critical facial regions. This integrated feature representation allows for a more comprehensive capture of the dynamic changes in MEs, enabling the model to more accurately recognize and distinguish between different MEs. Through the visualization of high-level feature maps, we have successfully validated the effectiveness of the GA module and the LB module in capturing emotion-related facial regions. To achieve better feature fusion, we designed the AFF module, which dynamically adjusts the fusion weights and strategies according to the characteristics of the input data, thereby maximizing the fusion effect. To address the class imbalance issue in ME databases, we introduced the CB-SCE loss. Experimental results demonstrate that the CB-SCE loss significantly improves performance in MER tasks and effectively mitigates the impact of class imbalance.

However, most current research relies on optical flow methods for processing ME data, which constrains the advancement of the MER field. In future research, we plan to reduce our reliance on optical flow estimation methods and explore adaptive motion description approaches suitable for end-to-end MER frameworks. Additionally, we intend to integrate multi-modal signals, such as speech and electroencephalograms, to further enhance the model’s performance and robustness.

## Figures and Tables

**Table 1:** Detailed information on the sample distributions for the SMIC-HS, CASME II, SAMM and Combined.

SMIC-HS [20]		CASME II [40]		SAMM [41]		Combined [48]	
Expression	Number	Expression	Number	Expression	Number	Expression	Number
Positive	51	Happiness	32	Happiness	26	Positive	109
Negative	70	Repression	27	Contempt	11	Negative	250
Surprise	43	Surprise	25	Surprise	15	Surprise	83
-	-	Disgust	62	Angry	56	-	-
-	-	Others	99	Others	27	-	-

**Table 2:** Comparison of results with state-of-the-art methods under SDE standard. The best results are shown in bold, and the second-best results are underlined.

Method	Year	Type	SMIC (3 Class)		CASME II (5 Class)		SAMM (5 Class)	
			ACC	F1	ACC	F1	ACC	F1
LBP-TOP [20]	2014	-	43.38	0.3421	39.68	0.3589	39.68	0.3589
Bi-WOOF [19]	2018	-	62.20	0.6200	58.85	0.6100	-	-
DSSN [26]	2019	CNN	63.41	0.6462	70.78	0.7297	57.35	0.4644
STRCN [25]	2019	CNN	53.1	0.514	56.0	0.542	54.5	0.492
GEME [28]	2021	CNN	64.63	0.6158	75.20	0.7354	55.88	0.4538
Later [31]	2022	ViT	73.17	0.7447	70.68	0.7106	-	-
SLSTT-LSTM [32]	2022	LSTM+ViT	75.00	0.740	75.81	0.753	72.39	0.640
FRL-DGT [12]	2023	ViT	-	-	75.70	0.748	-	-
SS-GN [30]	2023	GCN	-	-	72.90	0.716	75.17	**0.732**
C3DBed [34]	2023	CNN+ViT	78.04	0.7784	77.64	0.7520	75.73	0.7216
SRMCL [35]	2024	ViT	**78.98**	**0.7887**	83.20	0.8286	74.63	0.6599
MMTNet [36]	2025	ViT	78.05	0.7799	80.08	0.8007	75.00	0.6736
CSARNet [29]	2025	CNN	-	-	78.46	0.7665	**77.94**	0.6980
**GLFNet (ours)**	2025	CNN	78.05	0.7775	**84.10**	**0.8399**	**77.94**	0.7278

**Table 3:** Comparison of results with state-of-the-art methods under CDE standard. The best results are shown in bold, and the second-best results are underlined.

Method	Year	Type	Combined		SMIC		CASME II		SAMM	
			UF1	UAR	UF1	UAR	UF1	UAR	UF1	UAR
LBP-TOP [20]	2014	-	0.5882	0.5785	0.2000	0.5280	0.7026	0.7429	0.3954	0.4102
Bi-WOOF [19]	2018	-	0.6296	0.6227	0.5727	0.5829	0.7805	0.8026	0.5211	0.5139
STSTNet [6]	2019	CNN	0.7353	0.7605	0.6801	0.7013	0.8382	0.8686	0.6588	0.6810
AU-GCN [33]	2021	GCN+ViT	0.7914	0.7914	0.7651	0.7780	0.9071	0.8878	0.7392	0.7163
SLSTT-LSTM [32]	2022	LSTM+ViT	0.816	0.790	0.740	0.720	0.901	0.885	0.715	0.643
FeatRef [14]	2022	CNN	0.7838	0.7832	0.7011	0.7083	0.8915	0.8873	0.7372	0.7155
BDCNN [27]	2022	CNN	0.8509	0.8500	0.7859	0.7869	0.9501	0.9516	0.8538	0.8507
C3DBed [34]	2023	CNN+ViT	0.8075	0.8013	0.7760	0.7703	0.8978	0.8882	0.8126	0.8067
HTNet [4]	2023	ViT	0.8603	0.8475	0.8049	0.7905	0.9532	0.9516	0.8131	0.8124
SRMCL [35]	2024	ViT	0.8630	**0.8830**	0.7946	0.8053	0.9635	0.9649	0.8470	0.8866
MMTNet [36]	2025	ViT	0.8321	0.8233	0.8103	**0.8072**	0.8762	0.8690	0.7789	0.7552
CSARNet [29]	2025	CNN	0.8239	0.8300	0.7605	0.7639	0.9254	0.9298	0.7894	0.7924
**GLFNet (ours)**	2025	CNN	**0.8879**	0.8788	**0.8148**	0.8029	**0.9684**	**0.9753**	**0.8937**	**0.8931**

**Table 4:** Ablation Study of GLFNet Components on CASME II Dataset. The best results are highlighted in bold. In the table, “✓” denotes that the module is used, whereas “×” denotes that the module is not used.

GA	LB	AFF	CB-SCE	CASME II (5 Class)			
				ACC	F1	UF1	UAR
✓	×	×	×	79.92	0.7956	0.7783	0.7624
×	✓	×	×	81.17	0.8125	0.8083	0.8149
✓	✓	×	×	81.59	0.8155	0.8138	0.8043
✓	✓	✓	×	**84.10**	0.8391	0.8340	0.8330
✓	✓	×	✓	82.43	0.8261	0.8284	0.8314
✓	✓	✓	✓	**84.10**	**0.8399**	**0.8383**	**0.8535**

**Table 5:** Evaluation of backbone networks. The best results are highlighted in bold.

Method	CASME II (5 Class)			
	ACC	F1	UF1	UAR
ResNet-8	74.06	0.7278	0.6879	0.6732
ResNet-10	**76.15**	**0.7549**	0.7295	0.7233
ResNet-12	75.31	0.7491	0.7196	0.7243
ResNet-14	74.90	0.7416	0.7175	0.7178
ResNet-16	75.31	0.7475	0.7292	0.7120
ResNet-18	75.73	0.7539	**0.7445**	**0.7453**

**Table 6:** Evaluation of Parameters and Performance of GA and LB Modules and GLFNet Under Different Backbone Networks. The best results are highlighted in bold.

Method	Parameter	CASME II (5 Class)			
		ACC	F1	UF1	UAR
GA (ResNet-18)	27.57 M	76.99	0.7701	0.7808	0.7641
GA	13.09 M	79.92	0.7956	0.7783	0.7624
LB (ResNet-18)	52.36 M	78.66	0.7832	0.7549	0.7465
LB	23.66 M	81.17	0.8125	0.8083	0.8149
GLFNet(ResNet-18)	79.77 M	82.01	0.8195	0.8055	0.8164
GLFNet	36.97 M	**0.8410**	**0.8391**	**0.8340**	**0.8330**

**Table 7:** Evaluation of feature fusion methods. The best results are highlighted in bold.

Method	CASME II (5 Class)			
	ACC	F1	UF1	UAR
Concat	82.43	0.8261	0.8284	0.8314
Add	83.26	0.8332	0.8297	0.8286
Mul	82.01	0.8194	0.8156	0.8157
AFF	**84.10**	**0.8391**	**0.8340**	**0.8330**

**Table 8:** Evaluation of different loss functions. The best results are highlighted in bold.

Loss	CASME II (5 Class)			
	ACC	F1	UF1	UAR
SCE	**84.10**	0.8391	0.8340	0.8330
Focal	82.85	0.8271	0.8345	0.8465
CB-SCE	**84.10**	**0.8399**	**0.8383**	**0.8535**

**Table 9:** Comparison of the number of parameters and execution time between GLFNet and existing methods, where execution time refers to the average time required to process each sample during training and testing.

Method	Year	Type	Params (M)	Execution Time (S)
STSTNet [6]	2019	CNN	0.00167	10.04
DSSN [26]	2019	CNN	0.97	14.07
BDCNN [27]	2022	CNN	10.88	131.14
SLSTT-LSTM [32]	2022	ViT+LSTM	93.05	83.77
HTNet [4]	2023	ViT	149.57	137.26
SRMCL [35]	2024	ViT	342.15	169.13
GLFNet (ours)	2025	CNN	36.97	51.61

## Data Availability

The SMIC database used in this study was provided by the research team led by Li Xiaobai at the University of Oulu, Finland. Authorization is required to use this database. To obtain access, please email xiaobai.li@oulu.fi for permission. The CASME II database used in this study was provided by the research team led by Lan Fu at the Chinese Academy of Sciences. Authorization is required to use this database. To obtain access, please visit http://casme.psych.ac.cn/casme/e2 accessed on 1 August 2025, download and sign the license agreement, and send it to fuxl@psych.ac.cn. The SAMM database used in this study was provided by the research team led by Moi Hoon Yap at Manchester Metropolitan University. Authorization is required to use this database. To obtain access, please visit https://helward.mmu.ac.uk/STAFF/M.Yap/dataset.php accessed on 1 August 2025, download and sign the license agreement, and send it to m.yap@mmu.ac.uk. The data generated during this study are available upon request by contacting the corresponding author at yangwenzhong@xju.edu.cn.
